# Integrative bioinformatics and molecular analysis revealed the roles of *mTOR/S6K* Axis, *CASC15*, and *miR-30a-3p* in laryngeal squamous cell carcinoma

**DOI:** 10.1038/s41598-026-39618-w

**Published:** 2026-02-10

**Authors:** Najmeh parvaz, Payam Mohammadi, Maryam Lotfi, Mohammad Elahimanesh, Mohammad Reza Hajizadeh, Masoomeh Bakhshandeh, Mohammad Shabani, Mohammad Najafi

**Affiliations:** 1 Department of Biochemistry, School of Medicine, Iran University of Medical Sciences, Tehran, Iran; 2 Otorhinolaryngology Research Center, Amir Alam Hospital, Tehran University of Medical Sciences, Tehran, Iran; 3 Department of Clinical Biochemistry, School of Medicine, Rafsanjan University of Medical Sciences, Rafsanjan, Iran; 4 Molecular Medicine Research Center, Institute of Basic Medical Sciences Research, Rafsanjan University of Medical Sciences, Rafsanjan, Iran; 5 Microbial Biotechnology Research Center, Iran University of Medical Sciences, Tehran, Iran; 6 Department of Biochemistry, School of Medicine, Microbial Biotechnology Research Center, Iran University of Medical Sciences, Tehran, Iran

**Keywords:** LSCC, *mTOR* signaling pathway, miR-30a-3p, CASC15, Biomarkers, Cancer, Computational biology and bioinformatics, Genetics, Molecular biology, Oncology

## Abstract

Dysregulation of signaling pathways contributes substantially to the initiation and progression of laryngeal squamous cell carcinoma (LSCC). This study aimed to elucidate the *mTOR* and S6 kinase (*S6K*) gene expression levels, clinical significance, interplay of the *mTOR/ S6K* axis, *miR-30a-3p*, and the long non-coding RNA *CASC15* in LSCC using bioinformatics tools. RNA-seq datasets (TCGA and GEO) were analyzed through bioinformatics and network-based tools to identify candidate pathways, miRNAs, and lncRNAs using R and Cytoscape. *mTOR*, *S6K*, *miR-30a-3p*, and *CASC15* gene expression levels and *mTOR* and *S6K* protein values were measured in paired tumor (*n* = 54) and adjacent normal tissues (*n* = 54) using RT-qPCR and western blotting, respectively. Network analysis predicted that the *mTOR/S6K* regulatory axis is associated with *miR-30a-3p* and *CASC15*. The *mTOR* (*p* < 0.01), S6K (*p* < 0.0001), and *CASC15* (*p* = 0.021) gene expression levels were significantly upregulated, whereas *miR-30a-3p* was markedly downregulated (*p* < 0.0001). *miR-30a-3p* expression levels showed inversely correlated to *mTOR* (*p* = 0.05) and *S6K* (*p* = 0.01), while CASC15 correlated positively to both *mTOR* (*p* = 0.00) and S6K (*p* = 0.00). The *mTOR/S6K* signaling axis is markedly activated in LSCC. *CASC15* may act as an oncogenic lncRNA, whereas miR-30a-3p may function as a tumor-suppressive miRNA, suggesting their potential as molecular targets in LSCC.

**Supplementary Information:**

The online version contains supplementary material available at 10.1038/s41598-026-39618-w.

## Introduction

Laryngeal squamous cell carcinoma (LSCC) is one of the most common subtypes of head and neck neoplasms, with a rising incidence observed each year globally^1^. The pathogenesis of laryngeal cancer involves intricate interactions between genetic background and environmental risk factors^2^. Tobacco smoking, alcohol drinking, and human papillomavirus (HPV) infection are known as major risk factors for cancer development^3^. Despite significant advancements in therapeutic strategies for laryngeal cancer, including surgery, chemotherapy, radiotherapy, and immunotherapy, LSCC has a poor prognosis and a low survival rate. Therefore, finding reliable factors to determine molecular pathogenesis and improving therapy approaches is crucial^4^.

Many molecular signaling pathways related to cell proliferation, growth, and survival are reported to be altered in cancers^5^. One of the most crucial routes in cancer development and progression is the *PI3K/AKT/mTOR* signaling cascade^6^. *mTOR*, a downstream target of the *PI3K/Akt* pathway, plays an important role in regulating protein synthesis and breakdown in cancer cells by activating eukaryotic translation initiation factor 4E-binding protein 1 (*4E-BP1*) and S6 kinases (*S6Ks*)^7,8^. Abnormal modification of the mTOR signaling pathway has been reported in multiple cancers, including laryngeal cancer^4,9,10^. Integrin Subunit Alpha 5 (*ITGA5*), a target of *mTORC1*, potentially elevates LSCC progression^4^. On the other hand, inhibition of the *PI3K/Akt/mTOR* pathway has been reported to enhance apoptosis while suppressing proliferation, migration, invasion, and tumor growth in LSCC cell lines^10^. Moreover, tumor-associated antigens such as preferentially expressed antigen in melanoma (PRAME) have been found to promote proliferation, migration, invasion, and epithelial–mesenchymal transition (EMT), partly through activation of downstream *PI3K/AKT/mTOR* signaling^9^.

Besides genetic factors, various regulatory elements, including long noncoding RNAs (lncRNAs), circular RNAs, and microRNAs (miRNAs), control cellular biological activities^11^. In cancer, lncRNAs and miRNAs may act as either oncogenes or tumor suppressors, depending on their target genes^12^. LncRNAs facilitate tumorigenesis by altering molecular signaling pathways^13^. miRNAs are small, non-coding RNAs, typically 21–23 nucleotides in length, that regulate gene expression post-transcriptionally by binding to target mRNAs. Non-coding RNAs are known as gene regulators. The competitive endogenous RNA (ceRNA) hypothesis suggests that lncRNAs compete with mRNAs for binding to miRNAs, thereby serving as critical regulators of mRNA expression levels^14^. Although aberrant expression of multiple lncRNAs and miRNAs has been observed in various malignancies, including LSCC^15,16^, the clinical applicability remains constrained due to their undefined activities. Thus, understanding their functions is essential for developing novel diagnostic and therapeutic approaches.

Although *mTOR* signaling has been extensively studied in LSCC, its relationship with regulatory molecules such as *miR-30a-3p* and *CASC15* remains unclear. This study was conducted in two phases: first, bioinformatic analysis, followed by expression validation in clinical samples. The aim was to identify the signaling pathways implicated in the pathogenesis of laryngeal squamous cell carcinoma by analyzing RNA-sequencing datasets TCGA and GEO through systems biology and network-based techniques. Candidate miRNAs and lncRNAs associated with the mTOR signaling pathway were predicted through bioinformatic databases. Additionally, the expression levels of specific genes (*mTOR*, *S6K*), miRNA (*miR-30a-3p*), and lncRNA (CASC15) were analyzed in both healthy and tumor tissues.

## Materials and methods

### Signaling pathway network construction

The RNA-seq data related to laryngeal cancer were obtained from the TCGA (https://portal.gdc.cancer.gov) database. Genes demonstrating significant elevation (P-value < 0.05 and Log Fold Change (LFC) > 0.5) were selected to capture a broader set of upregulated genes contributing to signaling pathways (Supplement 1 A). Then, the gene collection was enriched using the KEGG database^15–17^ to identify the signaling pathway implicated in cancer. The PathIn online web tool (https://pathin.cing-big.hpcf.cyi.ac.cy) and Cytoscape (https://cytoscape.org) were used to construct a signaling pathway network. The nodes were represented signaling pathways and their sizes were determined by a score derived from (i) text mining using the PubTator3 web server (https://www.ncbi.nlm.nih.gov/research/pubtator3) (keywords: laryngeal cancer, larynx cancer, laryngeal squamous cell carcinoma, LSCC, names of signaling pathways) and (ii) the ratio of the number of genes identified in the analysis for each pathway to the total number of genes within the pathway (Supplement 1B). Additionally, edges indicated genes that are common to two connected biological signaling pathways. Thicker edges showed a higher quantity of shared genes between pathways.

### NcRNA prediction

The GEO database (https://www.ncbi.nlm.nih.gov/geo) was used to determine the miRNA expression profiles of LSCC (GSE136632, GSE132222). The expression profiles of lncRNAs were initially obtained from TCGA and GEO online databases (GSE133632, GSE142083) (Supplement 1 C, D, E). The differentially expressed miRNAs (DE miRNAs) and lncRNAs (DE lncRNAs) were determined using the DESeq2 package in R (|LFC > 1|, adjusted *P* < 0.05).

Moreover, miRNA−gene data were collected from miRTarBase (https://mirtarbase.cuhk.edu.cn), Starbase (https://rnasysu.com/encori), DIANA (https://diana.e-ce.uth.gr), TargetScan (https://www.targetscan.org), miRWalk (http://mirwalk.umm.uni-heidelberg.de), miRDB (https://mirdb.org), and miRmap (https://mirmap.ezlab.org). DIANA was conducted to predict lncRNA−miRNA associations (Supplement 1 C, D, E). A ceRNA association network was subsequently constructed using Cytoscape (version v3.10.3, https://cytoscape.org).

### Human tissue specimens

A total of 54 laryngeal squamous cell carcinoma tissues and corresponding adjacent normal tissues were obtained from patients diagnosed with laryngeal carcinoma at pathological stages III and IV from the tumor bank of the Otorhinolaryngology Research Center at Amir Alam Hospital in Tehran, Iran. Informed consent was obtained from all patients. The tissues were assessed by a pathologist and cryopreserved at -80^°C^. The current study received approval from the Ethics Committee of the Iran University of Medical Sciences (IR.IUMS.FMD.REC.1402.508). All methods were performed in accordance with the relevant guidelines and regulations.

### Quantitative reverse transcription polymerase chain reaction (RT-qPCR)

Total RNA was isolated from all tissues using the protocol of the Total RNA Isolation Kit (BIO BASIC CANADA Inc., Canada). RNA was reverse-transcribed into complementary DNA (cDNA) using the cDNA Synthesis Kit (Zist Virayesh cDNA synthesis kit, Iran) according to the manufacturer’s instructions. One designated stem-loop was employed for cDNA synthesis from mature miRNA (Table 1).

RT-qPCR was subsequently conducted using the Real-Time PCR Detection System (StepOne) using the Zist Virayesh SYBR qPCR Master Mix (Tehran, Iran). The reaction conditions were performed at 95 °C for 10 min, followed by 40 cycles (95 °C for 15 s, and annealing temperature for 30 s). Melt curve analysis was performed by gradually increasing the temperature from 55 °C to 95 °C in 0.5 °C increments, with continuous monitoring of SYBR Green fluorescence. The 2^−ΔΔCt^ method was used to estimate the gene, miRNA, and lncRNA expression levels. Glyceraldehyde-3-phosphate dehydrogenase (*GAPDH*) and *U6* were used as housekeeping genes for mRNAs and miRNAs, respectively. Primers are shown in Table 1.

**Table 1:** Primer sequences used for RT-qPCR.

Gene/ncRNA/Loop	Primer sequences (5’-3’)	Annealing temperature (°C)
*mTOR*	GCGTCCCTACCTTCTTCTTCCAG GTCTCATCAAATCCCTTCTCTGCTTC	67
*S6K*	CAGTGAAAGTGCCAATCAGGTCT GTGTCTGAGGATTTGCTGTGCTG	67
*miR30a-3p*	CCACGGGCTTTCAGTCGGATGT CGTTGGCTCTGGTGCTGGGT	63
*miR30a-3p*-loop	CGTTGGCTCTGGTGCTGGGTCCGAGGTATTCGCACCAGAGCCAACGGCTGCAA	
*CASC15*	AGTATGGGCAGGCTTGATTTCTT TCTTCTTGGCTGAGGTTGTGTTG	60
*GAPDH*	CATGAGAAGTATGACAACAGCC AGTCCTTCCACGATACCAAAGT	58
*U6*	TTGGAACGATACGGAGAAGATTAGC TATGGAACGCTTCACGAATTTGC	68

### Western blot analysis

Tissue samples were lysed with radioimmunoprecipitation assay buffer (RIPA), and proteins were extracted and quantified using the Bradford assay (Protein Quantification kit (DB0017, DNA bioTech, Iran)). Lysates (20 µg) were subjected to sodium dodecyl sulfate–polyacrylamide gel electrophoresis (SDS-PAGE) and subsequently transferred to polyvinylidene difluoride (PVDF) membranes (Cat No: 162-017777; Bio-Rad Laboratories, CA, USA). Membranes were blocked using 5% bovine serum albumin (BSA), then incubated with primary antibodies and horseradish peroxidase-conjugated secondary antibodies (HRP) (Cat No: ab6721; Abcam, 1:10000 dilution). Enhanced chemiluminescence was used to visualize the protein bands, and densitometry was used to quantify them. The protein expression levels were normalized to *GAPDH*. The primary antibodies included *mTOR* (Cat No: 2971 S, Cell Signaling, 1:1000), *S6K* (Cat No: M01475-1, Boster Bio, 1:1000), and *GAPDH* (Cat No: ab8245, Abcam, 1:2500). ImageJ software (1.52v, NIH) was used to estimate the protein values.

### Hematoxylin and Eosin (HE) staining

HE staining was used for the histopathology. 6-µm-thick slices obtained from paraffin-embedded tissues were stained with hematoxylin and eosin for microscopic examination.

### Statistical analyses

Statistical Package for the Social Sciences (SPSS) version 26.0 and GraphPad Prism 8.0 were used for data analysis. The gene data were evaluated on a normalization test. The Wilcoxon test was performed to compare differences between the LSCC and normal tissues. The Mann-Whitney U test was used to compare gene data between clinicopathological features in LSCC tissue samples. Correlations between factors were estimated using the Spearman. The gene expression data were replicated three times. Significance was set at < 0.05.

## Results

### *mTOR *signaling pathway was predicted from the signaling pathway network

**Fig. 1:**
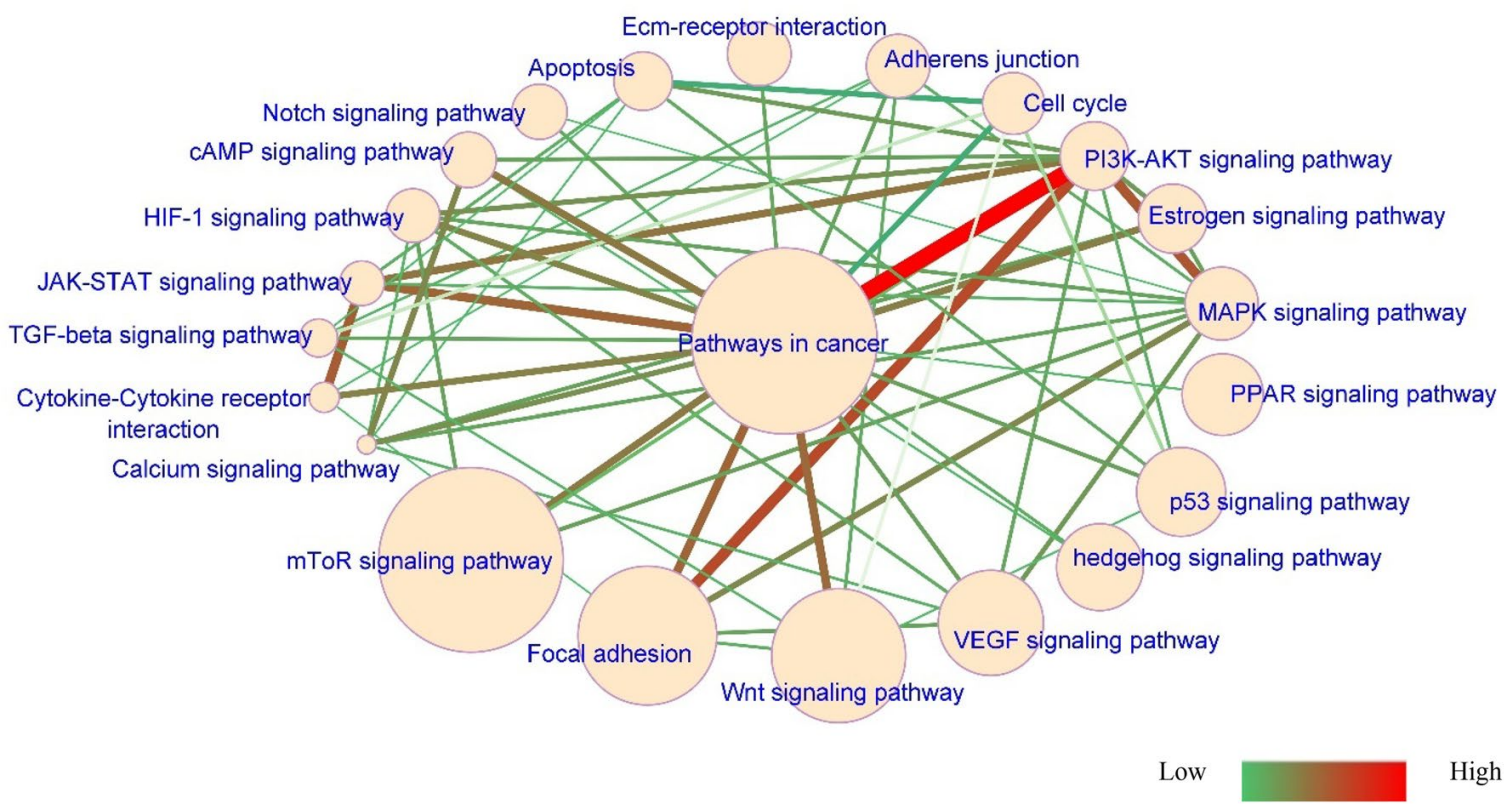
Signaling pathways network. The network comprises 22 nodes. Each node shows a signaling pathway, and its size is estimated based on the number of genes upregulated significantly in LSCC. The *mTOR* signaling pathway has the largest node size. Each edge shows genes that are shared between two biological signaling pathways. Thicker edges show higher scores, and thinner edges represent lower scores.

Data analysis from the TCGA database indicated that the “Pathways in cancer” has a substantial role in laryngeal cancer. This circuit includes various cancer-associated signaling cascades (Fig. 1). Some of the high edge signaling pathways are the *PI3K/AKT* pathway (*N* = 11, edge score: 13.75), *MAPK* pathway (*N* = 10, edge score: 12.85), focal adhesion (*N* = 7, edge score: 8.68), cell cycle (*N* = 15, edge score: 6.65), and *Wnt* pathway (*N* = 6, edge score: 5.30). The prominent nodes were *mTOR* (node size score: 8.13), focal adhesion (node size score: 6.48), and the *Wnt* pathway (node size score: 6.31). The mTOR signaling pathway had the largest node sizes (8.13) and was interconnected with other significant pathways. This evidence indicates its pivotal significance in laryngeal carcinoma. Consequently, the *mTOR* pathway was chosen for subsequent experimental exploration in the following phases of this study.

### *miR3a-3p* and *CASC15* were predicted to be involved in the gene/miRNA/lncRNA network

**Fig. 2:**
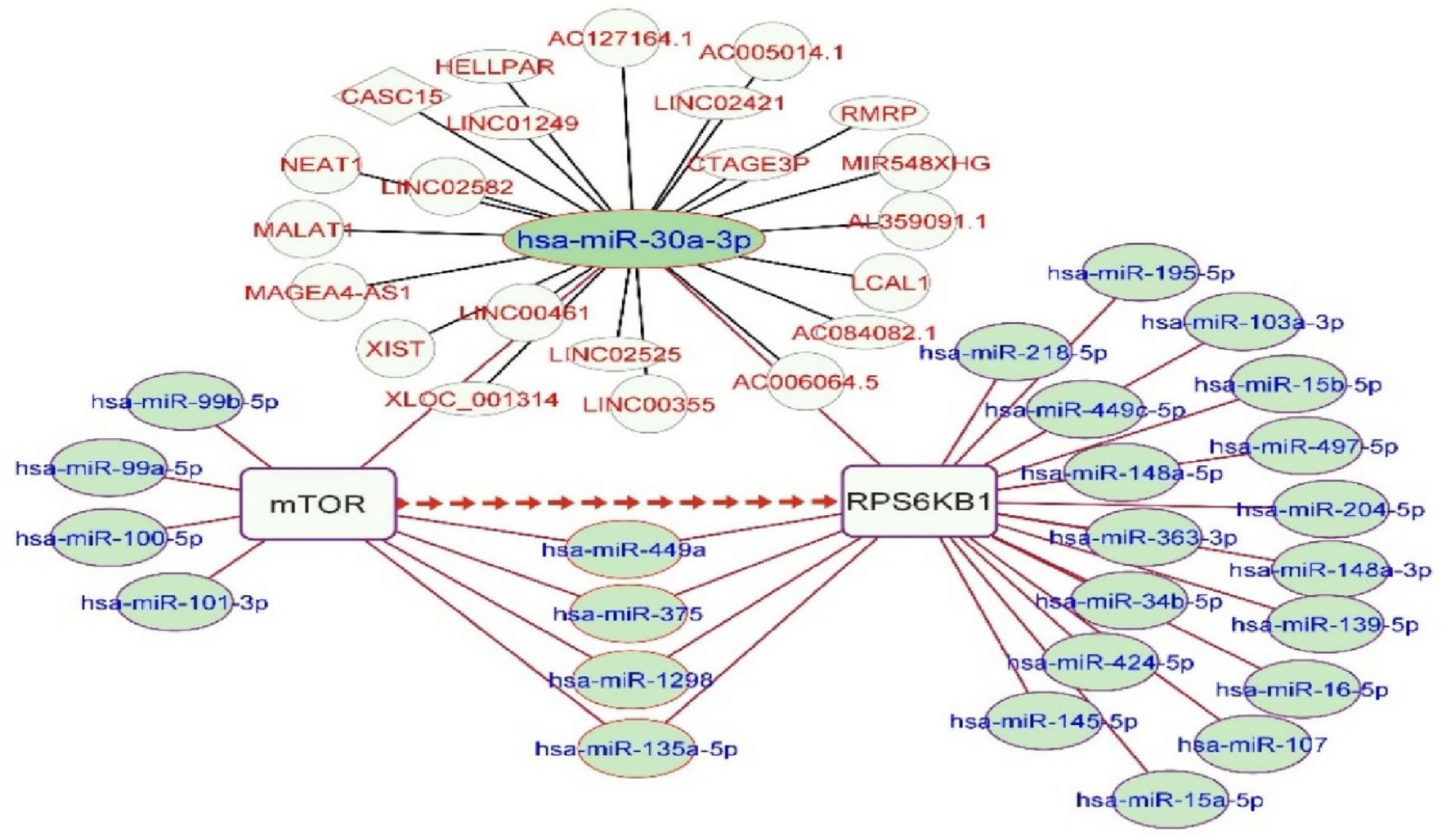
Genes/miRNAs/lncRNAs network. The red arrow indicates the direction of the *mTOR/S6K* axis. Green nodes denote common miRNAs that target *mTOR* and *S6K*. The lncRNAs linked to *miR-30a-3p*. Nodes represent miRNAs and lncRNAs associated with the *mTOR/S6K* axis. The edges represent predicted regulatory interactions based on TCGA, GEO, and other databases.

The *mTOR/S6K* axis in the *mTOR* pathway was selected to construct the gene/lncRNA/miRNA network. miRNAs showing decreased expression in tumor tissues were identified in GEO datasets. Data were aggregated from different established databases to discover putative regulatory miRNAs linked to both *mTOR* and *S6K*. Five miRNAs were frequently associated with both genes, including *miR-30a-3p*, *miR-449a*, *miR-375*, *miR-1298*, and *miR-135a* (fold change > 3). Among these, *miR-30a-3p* was designated as the principal candidate for subsequent study (Fig. 2).

LncRNAs having a score > 5.5 were selected to form the miRNA-lncRNA association network. *CASC15* and *LINC00461* were considered those that consistently elevated in both TCGA and GSE datasets. Additionally, the DIANA database forecasted an association between them and miR-30a-3p (Fig. 2).

### The *mTOR*, *S6K*, and *CASC15 *gene expression levels increased, while *miR-30a-3p *decreased in LSCC tissues

**Fig. 3:**
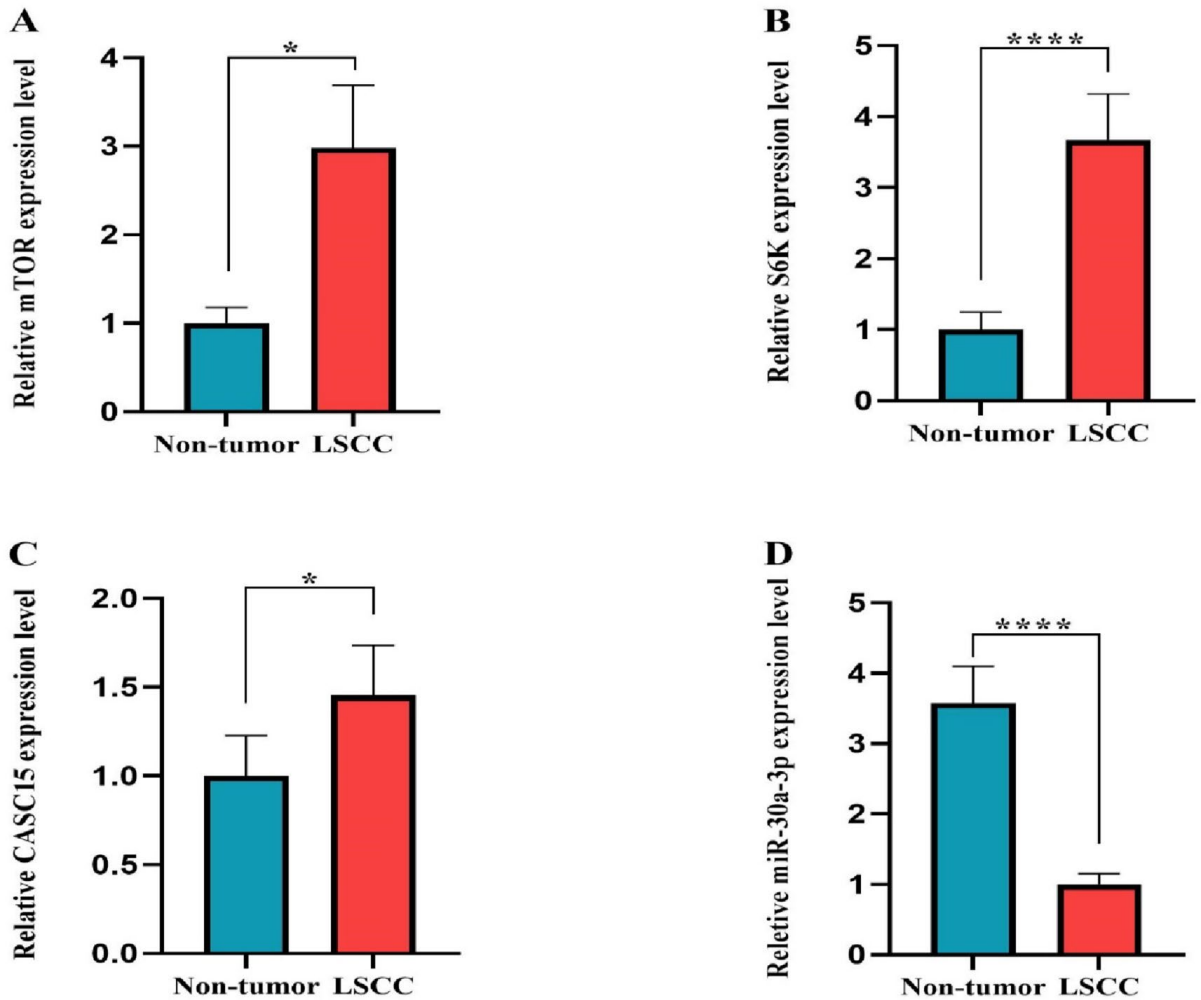
The gene expression levels of *mTOR*, *S6K*, lncRNA *CASC15*, and *miR-30a-3p*. *mTOR* (A), *S6K* (B), and *CASC15* (C) genes are upregulated in LSCC tissues as compared to non-tumor tissues. *miR-30a-3p* (D) expression decreased in LSCC tissues. All data are expressed as mean ± SEM. (* *p* < 0.05 and **** *p* < 0.0001).

According to gene expression analysis, *mTOR* and *S6K* expression levels were significantly upregulated in laryngeal cancer tissues compared with adjacent normal tissues (*p* = 0.010) (Fig. 3A) and (*P* < 0.0001) (Fig. 3B), respectively. Similarly, the expression of long non-coding RNA *CASC15* (*P* = 0.021) (Fig. 3C) was also elevated in tumor samples. In contrast, the *miR-30a* expression levels were markedly downregulated in cancerous tissues (*P* < 0.0001) (Fig. 3D).

### The *mTOR* and *S6K* protein expression levels increased in LSCC tissues

**Fig. 4:**
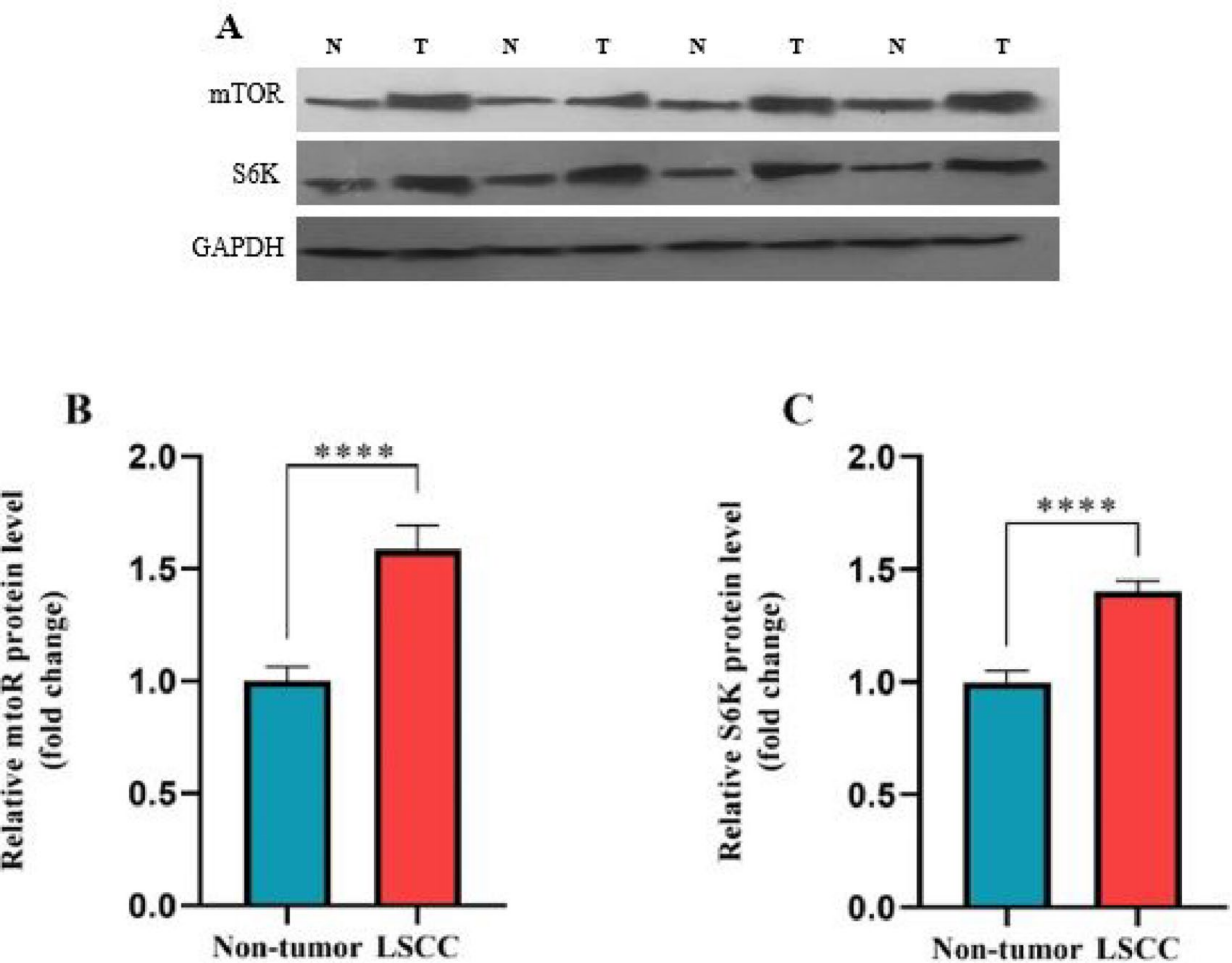
The protein expression levels of *mTOR* and *S6K* in the study population. Western blot image, N: normal tissues; T: Tumor (**A**). The *mTOR* (**B**) and *S6K* (**C**) protein levels are upregulated in LSCC tissues compared with non-tumor tissues. All data are expressed as mean ± SEM. (**** *P* < 0.0001).

The protein expression levels of *mTOR* and *S6K* elevated significantly in LSCC tissues compared with adjacent non-cancerous tissues (Fig. 4, Supplement 2 (Uncropped images)). These results were in line with the gene expression data.

### The *mTOR* and *CASC15* expression levels were related to smoking through clinicopathological characteristics

**Fig. 5:**
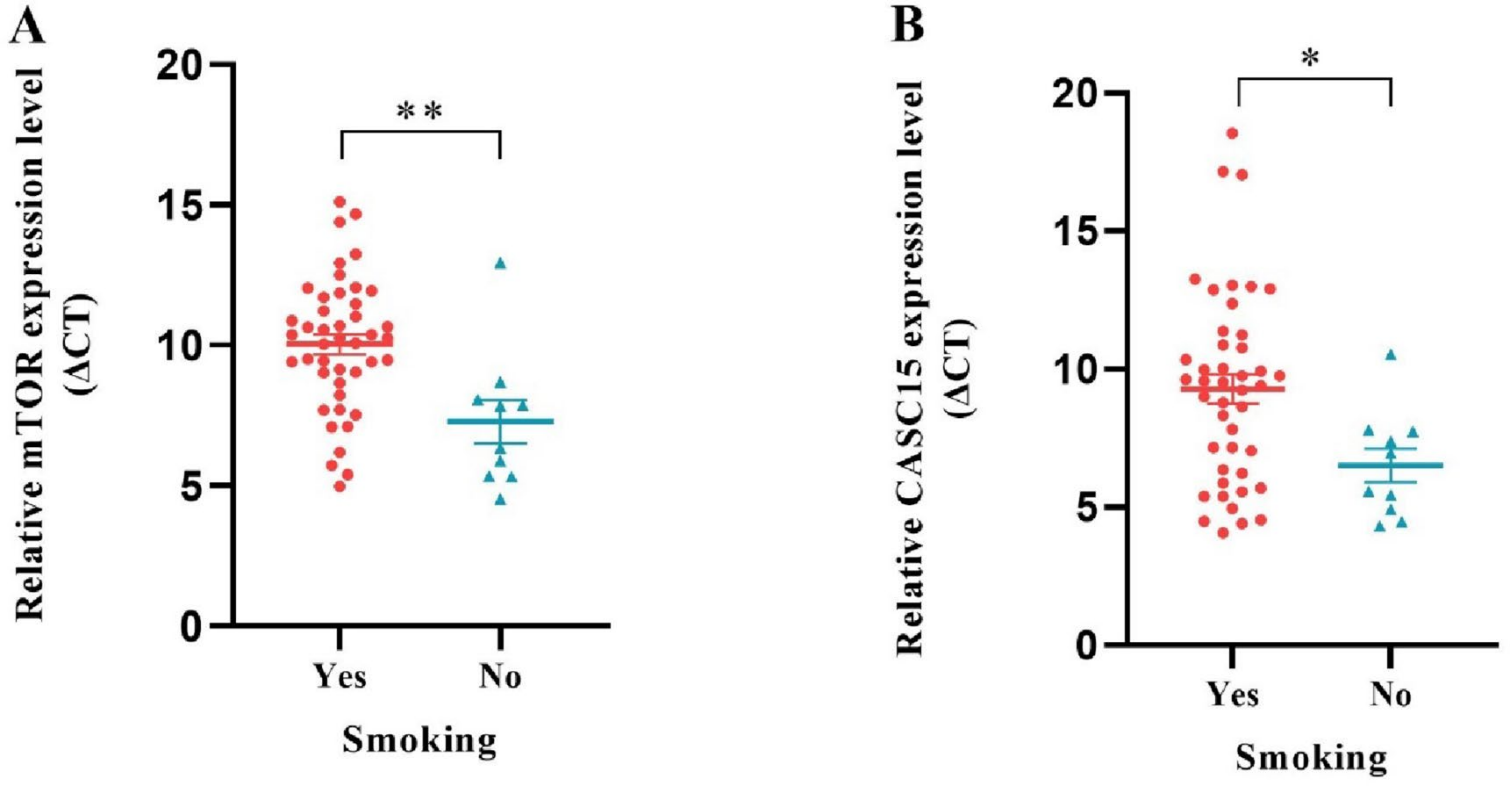
Smoking and gene expression levels. *mTOR* (**A**) and *CASC15* (**B**) expression levels were significantly elevated in smokers compared to nonsmokers (**p = 0.002, *p = 0.019).

Table 2 summarizes the clinicopathological characteristics of the patients. There was a significant association between smoking and increased expression of *mTOR* (Fig. 5A) and *CASC15* (Fig. 5B). No significant associations were observed between *mTOR*, *S6K*, *miR-30a-3p*, *CASC15*, and other clinicopathological factors.

**Table 2:** Relationships between *mTOR*, *S6K*, *miR-30a-3p*, and *CASC15* expression levels and clinicopathological features in LSCC patients.

Characteristic	Category	Frequency (Percent)	mTOR (*p* value)	S6K (*p* value)	miR-30a-3p (*p* value)	CASC15 (*p* value)
Age	≤ 60 > 60	37 (31.5%) 17 (68.5%)	0.425	0.127	0.299	0.726
T stage	III IV	23 (42.6%) 31 (57.4%)	0.515	0.343	0.530	0.083
Lymph node metastasis	0 1 2 3	35 (64.8%) 5 (9.3%) 11 (20.4%) 3 (5.6%)	0.407	0.527	0.392	0.558
Differentiation of cancer tissue	Well Moderate	35 (64.8%) 19 (35.2%)	0.303	0.937	0.153	0.726
Thyroid cartilage involvement	Yes No	31 (57.4%) 23 (42.6%)	0.515	0.343	0.530	0.083
Anterior commissure involvement	Yes No	26 (51.9%) 28 (48.1%)	0.678	0.588	0.496	0.946
Extra laryngeal soft tissues invasion	Yes No	22 (40.7%) 32 (59.3%)	0.761	0.226	0.358	0.165
Lymphovascular invasion	Yes No	16 (29.6%) 38 (70.4%)	0.092	0.227	0.086	0.774
Perineural invasion	Yes No	18 (33.3%) 36 (66.7%)	0.306	0.194	0.422	0.174
Epiglottis invasion	Yes No	35 (64.8%) 19 (35.2%)	0.697	0.695	0.450	0.773
Adjuvant therapy	Yes No	11 (20.4%) 43(79.6%)	0.929	0.692	0.181	0.525
Smoking	Yes No	44 (81.5%) 10 (18.5%)	0.002	0.360	0.408	0.019
Alcohol consumption	Yes No	4 (7.4%) 50 (92.6%)	0.826	0.608	0.616	0.915
Narcotics consumption	Yes No	23 (42.6%) 31 (57.4%)	0.717	0.973	0.725	0.425

### The *miR-30a-3p* exhibited an inverse correlation with *mTOR* and *S6K* expression levels, while *CASC15* showed a positive correlation with *mTOR* and *S6K* expression levels

Partial correlations were identified between *mTOR*, *S6K*, *miR-30a-3p*, and *CASC15* by controlling for variables including adjuvant therapy, smoking, alcohol consumption, and narcotics consumption. A negative correlation was observed between *mTOR* and *miR-30a-3p* (*r*=-0.193, *p* = 0.05). Similarly, *S6K* expression showed a significant negative correlation with *miR-30a-3p* (*r*=-0.223, *p* = 0.01). In contrast, *CASC15* exhibited significantly positive correlations with both *mTOR* (r = + 0.402, *p* = 0.000) and *S6K* (r = + 0.309, *p* = 0.001). A positive association between *mTOR* and *S6K* was detected (r = + 0.064, *p* = 0.51). Furthermore, *miR-30a-3p* and *CASC15* displayed a negative correlation (*r*=-0.024, *p* = 0.80) (Table 3).

**Table 3:** Correlations between *mTOR*, *S6K*, *miR-30a-3p* and *CASC15* gene expression levels.

Parameters		Spearman’s rho*	Confidence interval**	*p*-value
*mTOR*	*miR-30a-3p*	−0.193	−0.352 to −0.014	0.050
*S6K*	*miR-30a-3p*	−-0.233	−0.349 to −0.114	0.010
*CASC15*	*mTOR*	+ 0.402	0.156 to 0.615	< 0.001
*CASC15*	*S6K*	+ 0.309	0.087 to 0.525	0.001
*mTOR*	*S6K*	+ 0.064	−0.101 to 0.300	0.510
*CASC15*	*miR-30a-3p*	−0.024	−0.298 to 0.243	0.800

*Spearman’s rho values are presented as effect sizes. **Accompanied by 95%.

### 3.7 histopathology results

**Fig. 6:**
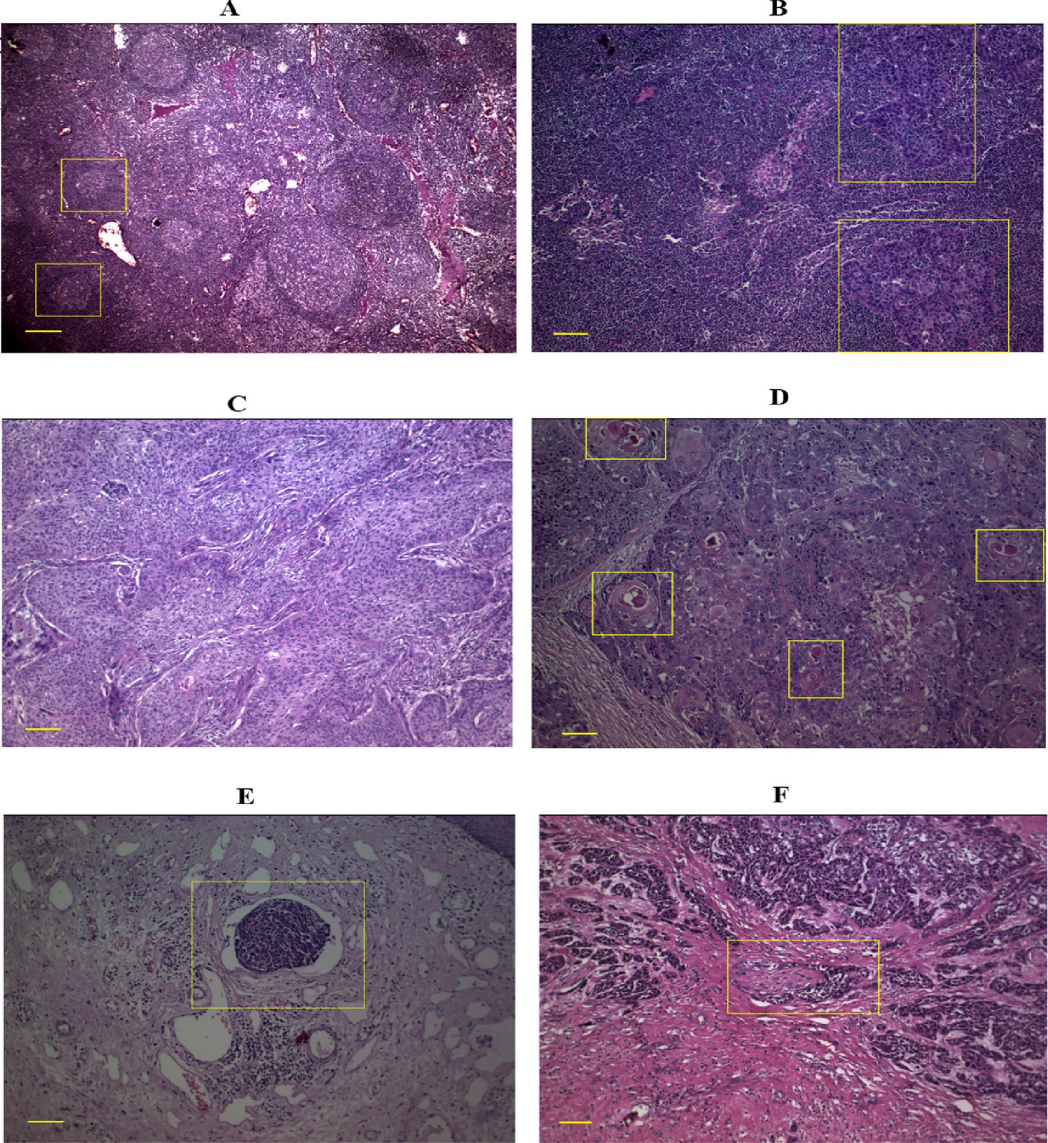
**A**: Normal lymph node (Scale bar: 0.025 mm, Magnification: 4x, Yellow rectangle: germinal center), **B**: lymph node metastasis (Scale bar: 0.1 mm, Magnification: 10x, Yellow rectangle: tumoral cell), **C**: Moderately differentiated (Scale bar: 0.1 mm, Magnification: 10x), **D**: Well differentiated (Scale bar: 0.1 mm, Magnification: 10x, Yellow rectangle: keratin pearl), **E**: Lymphovascular invasion (Scale bar: 0.1 mm, Magnification: 10x, Yellow rectangle: LVI), **F**: Perineural invasion (Scale bar: 0.1 mm, Magnification: 10x, Yellow rectangle: PNI).

Lymph node metastasis was observed in 35.2% of patients (Fig. 6A, B). 35.2% and 64.8% were moderately and well-differentiated tumors, respectively (Fig. 6C, D). Lymphovascular invasion (LVI) and perineural invasion (PNI) occurred in 29.6% and 33.3% of cases, respectively (Fig. 6E, F).

## Discussion

The present study aims to elucidate molecular alterations in laryngeal squamous cell carcinomas, focusing on the *mTOR/S6K* signaling axis and its potential relationship with *miR-30a-3p* and *CASC15*. Bioinformatics analysis identified the *mTOR* pathway as the main focus of the current research; however, other signaling pathways, including focal adhesion and *Wnt* signaling pathways, were also highly active in LSCC. The *mTOR/S6K* axis plays a crucial role in regulating protein synthesis during cell growth. In tumor cells, aberrantly active *mTOR* transmits signals that promote tumor cell proliferation, metastasis, and invasion of adjacent healthy tissues^18^. The activation of this pathway has been documented in numerous malignancies, including LSCC^13,19^, hepatocarcinoma^20,21^, and lung cancer^22^. A study reported that inhibition of the *PI3K/Akt/mTOR* pathway decreases phosphorylation of key components, including p-*Akt*, p-*p70S6K*, p-*4E-BP1*, and p-*S6RP*, resulting in enhanced apoptosis and reduced proliferation, migration, invasion, and tumor growth in LSCC cell lines^10^.

The decreased *miR-30a-3p* expression levels in tumor tissues suggested its potential role in impeding the progression of laryngeal cancer. *miR-30a-3p* has been reported to act as a tumor suppressor in various cancer types, such as laryngeal cancer^23^, bladder cancer^24^, gastric cancer^25^, and lung cancer^26^. *miR-30a-3p* targets DNA methyltransferase 3a (*DNMT3a*) and suppresses proliferation and invasion in LSCC and other cancer cells^23,27,28^. Furthermore, reduced levels of *miR-30a-3p* are associated with poor prognosis in head and neck squamous cell carcinomas^29^. Our results, in agreement with these studies, suggested that the suppressive effect of *miR-30a-3p* may be mediated by inhibiting key genes such as *mTOR* and *S6K*.

22 lncRNAs were associated with *miR-30a-3p* in the gene/miRNA/lncRNA network. According to bioinformatics analysis, *CASC15* was a candidate. Both bioinformatics and experimental analyses indicated the increased *CASC15* expression levels in tumor tissues. *CASC15* has been reported to have dual roles, functioning as a tumor suppressor in neuroblastoma^30^ and as an oncogene in colon^31^, lung^32^, gastric^33^, and hepatocellular carcinoma^34^. It sponged *miR-365* and caused the overexpression of cyclin D1 in laryngeal cancer^35^. It also facilitated cellular proliferation, motility, invasion, and EMT in gastric cancer via the *EZH2/WDR5–CDKN1A* axis and sponging *miR-33a-5p* to elevate *ZEB1*^36^. Additionally, the elevated *CASC15* expression levels in cervical cancer correlated with advanced stages and metastasis^37^. Bioinformatics analysis suggested an association between *CASC15* and *miR‑30a‑3p*, and experimental results were consistent, showing an inverse change in their expression levels without a significant correlation in LSCC. These changes may occur independently. Moreover, *miR‑30a‑3p* might act as an anti‑miRNA or suppresses transcription factors that normally inhibit *CASC15*. Further studies, such as RNA immunoprecipitation (RIP/RIP-seq), are required to clarify these mechanisms.

The concurrent overexpression of *CASC15*, *mTOR*, and *S6K* in tumor tissues enhanced intriguing possibilities about their functional relationships. The data suggested a potential modulatory role for *CASC15* in the *mTOR* pathway, potentially functioning as a competing endogenous RNA or via other regulatory mechanisms, as observed for other microRNAs such as *miR-7151-5p*^38^, *miR-766*^32^, and *miR-130b-3p*^39^. Moreover, the results showed a significant inverse correlation between *miR-30a-3p* expression and both *mTOR* and *S6K* levels in LSCC. This finding suggests that *miR-30a-3p* may play a regulatory role for the *mTOR/S6K* axis.

The clinicopathological correlations revealed the increased expression levels of *CASC15* and *mTOR* in smokers relative to non-smokers, aligning with documented associations between tobacco use and laryngeal cancer. It has been observed that *mTOR* is overexpressed in smokers with gastric cancer^40^. Furthermore, bladder cell growth and proliferation were inhibited after exposure to nicotine through activation of the *PI3K/Akt/mTOR* pathway^41^. In laryngeal cancer patients with a history of smoking, an increased expression level of nuclear paraspeckle assembly transcript 1 (*NEAT1*) has been documented^42^. These observations suggest that tobacco-related carcinogenic effects may entail the activation of signaling pathways and lncRNAs. However, these findings are limited by a sample size and must be confirmed in a larger population.

## Conclusion

This research delineated a molecular approach involving *mTOR*, *S6K*, *miR-30a-3p*, and *CASC15* in laryngeal squamous cell carcinoma. The results yielded significant insights; however, the bioinformatics analysis suggested other ncRNAs and signaling pathways to investigate LSCC molecular pathogenesis.

The study was limited by its small sample size, and the functional relationships between genes need to be experimentally validated across all pathological stages of laryngeal squamous cell carcinoma (LSCC) in multi-center cohorts. Furthermore, future research should focus on investigating the mechanistic roles of *CASC15* and *miR-30a-3p* in regulating the *mTOR/S6K* signaling axis in laryngeal cancer cells.

## Supplementary Information

Below is the link to the electronic supplementary material.

Supplementary Material 1

Supplementary Material 2

## Data Availability

The data are available from the corresponding author on reasonable request.

## Abbreviations

LSCC: Laryngeal Squamous Cell Carcinoma
HPV: Human Papillomavirus
4E-BP1: Eukaryotic Translation Initiation Factor 4E-Binding Protein 1
S6K: S6 Kinase
ITGA5: Integrin Subunit Alpha
PRAME: Preferentially Expressed Antigen in Melanoma
EMT: Epithelial-Mesenchymal Transition
lncRNAs: Long Non-coding RNAs
miRNAs: MicroRNAs
ceRNA: Competing Endogenous RNA
qRT-PCR: Quantitative Reverse Transcription Polymerase Chain Reaction
cDNA: Complementary DNA
GAPDH: Glyceraldehyde-3-Phosphate Dehydrogenase
RIPA: Radioimmunoprecipitation Assay
SDS-PAGE: Sodium Dodecyl Sulfate Polyacrylamide Gel Electrophoresis
PVDF: Polyvinylidene Difluoride
BSA: Bovine Serum Albumin
HRP: Horseradish Peroxidase
LVI: Lymphovascular Invasion
PNI: Perineural Invasion
DNMT3a: DNA Methyltransferase 3 Alpha
EMT: Epithelial-Mesenchymal Transition
CDKN1A: Cyclin-Dependent Kinase Inhibitor 1 A
EZH2: Enhancer of Zeste Homolog 2
WDR5: WD Repeat Domain 5
ZEB1: Zinc Finger E-box Binding Homeobox 1
NEAT1: Nuclear Paraspeckle Assembly Transcript 1
