# Supplementary material for: Integrative bioinformatics and molecular analysis revealed the roles of mTOR/S6K Axis, CASC15, and miR-30a-3p in laryngeal squamous cell carcinoma
Source: Sci Rep. 2026 Feb 10;16:8082. doi: 10.1038/s41598-026-39618-w (PMC12960688; doi:10.1038/s41598-026-39618-w)
Supplement: Supplementary file 2 — Supplementary Material 2 [file 41598_2026_39618_MOESM2_ESM.docx]

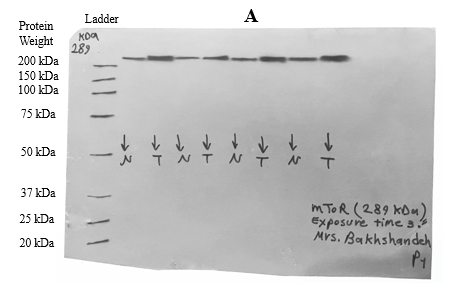

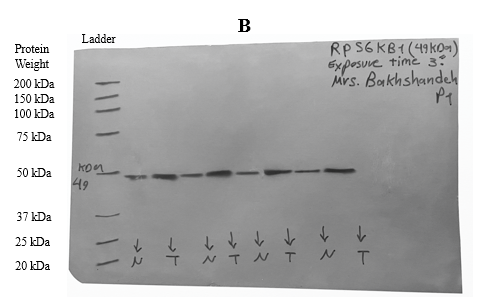

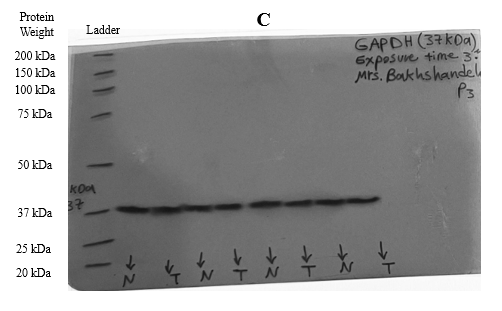


**Supplementary 2: Uncropped western blot Images**. mTOR (A) and S6K (B) protein expression levels **in tumor and adjacent non-tumor tissue samples from patients diagnosed with laryngeal squamous cell carcinoma (LSCC)**. GAPDH was used as a loading control to ensure equal protein loading across samples (C).N and T describe normal tissue (**non-tumor tissue**) and tumor tissue respectively.
